# Supplementary figures and images for: Long-Term Outcomes after Multimodal Treatment for Clival Chordoma: Efficacy of the Endonasal Transclival Approach with Early Adjuvant Radiation Therapy
Source: J Clin Med. 2023 Jul 3;12(13):4460. doi: 10.3390/jcm12134460 (PMC10342406; doi:10.3390/jcm12134460)

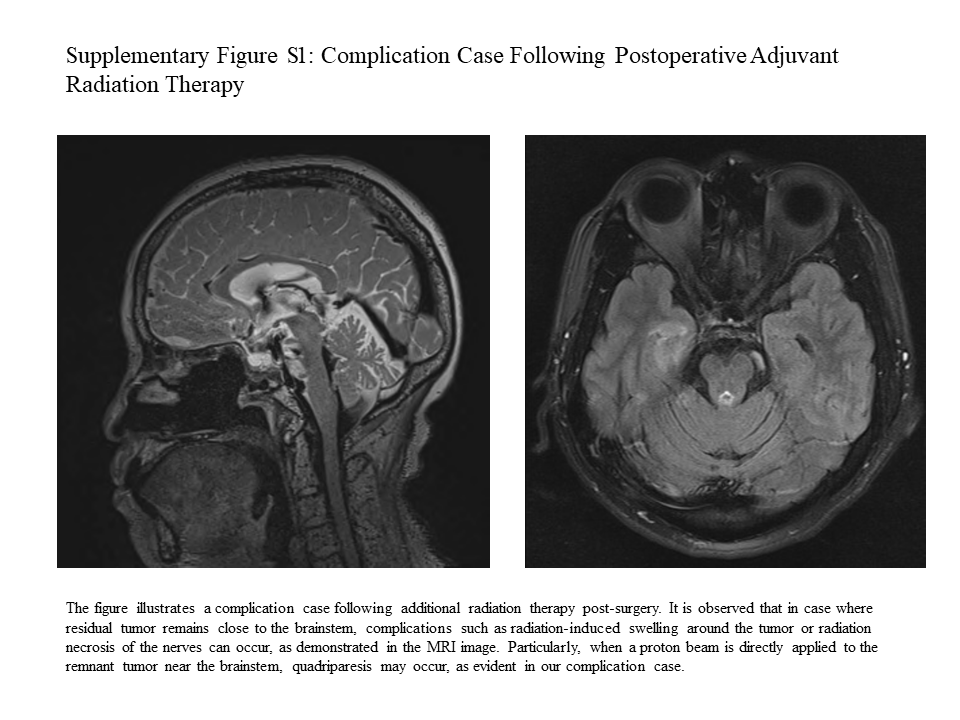

Supplement: Supplementary file 1 [file jcm-12-04460-s001.zip › jcm-2482807-supplementary.tif]
